# α-Synuclein colocalizes with AP180 and affects the size of clathrin lattices

**DOI:** 10.1016/j.jbc.2023.105091

**Published:** 2023-07-28

**Authors:** Karina J. Vargas, P.L. Colosi, Eric Girardi, Jae-Min Park, Leah E. Harmon, Sreeganga S. Chandra

**Affiliations:** 1 Departments of Neurology and Neuroscience, Yale University, New Haven, Connecticut, USA; 2 Marine Biological Laboratory, Woods Hole, Massachusetts, USA; 3 Department of Cell Biology, University of Pittsburgh, Pittsburgh, Pennsylvania, USA; 4 PREP Program, Yale University, New Haven, Connecticut, USA

**Keywords:** electron microscopy, lipid monolayer assay, membrane curvature, synaptic vesicle endocytosis, PI(4,5)P2

## Abstract

α-Synuclein and family members β- and γ-synuclein are presynaptic proteins that sense and generate membrane curvature, properties important for synaptic vesicle (SV) cycling. αβγ-synuclein triple knockout neurons exhibit SV endocytosis deficits. Here, we investigated if α-synuclein affects clathrin assembly *in vitro*. Visualizing clathrin assembly on membranes using a lipid monolayer system revealed that α-synuclein increases clathrin lattices size and curvature. On cell membranes, we observe that α-synuclein is colocalized with clathrin and its adapter AP180 in a concentric ring pattern. Clathrin puncta that contain both α-synuclein and AP180 were significantly larger than clathrin puncta containing either protein alone. We determined that this effect occurs in part through colocalization of α-synuclein with the phospholipid PI(4,5)P2 in the membrane. Immuno-electron microscopy (EM) of synaptosomes uncovered that α-synuclein relocalizes from SVs to the presynaptic membrane upon stimulation, positioning α-synuclein to function on presynaptic membranes during or after stimulation. Additionally, we show that deletion of synucleins impacts brain-derived clathrin-coated vesicle size. Thus, α-synuclein affects the size and curvature of clathrin structures on membranes and functions as an endocytic accessory protein.

α-Synuclein became a principal focus of neurodegeneration research when it was identified as the major constituent of Lewy Bodies, pathological protein aggregates found in the brains of Parkinson’s Disease (PD) patients (1). The importance of α-synuclein to PD pathogenesis was further underscored by the identification of families with Mendelian forms of PD that arise from point mutations and gene multiplications of *SNCA*, the α-synuclein gene (2, 3, 4, 5, 6, 7). Genome-wide association studies demonstrate that sequence variants in *SNCA* are associated with sporadic PD (8, 9). Additionally, Lewy body pathology is observed in several other neurodegenerative diseases, for instance, Lewy body dementia and multiple system atrophy, collectively known as synucleinopathies (10). Based on these observations, many current therapeutic strategies for PD, and more broadly synucleinopathies, are focused on eliminating or reducing α-synuclein levels in the brain (11, 12). Therefore, there is a growing interest in elucidating the underlying physiological functions of α-synuclein and how loss of α-synuclein impacts neuronal physiology.

α-Synuclein was initially discovered as a synaptic vesicle (SV)-associated protein in the electric organ of *Torpedo* (13), suggesting its physiological function(s) are linked to the SV cycle. Indeed, α-synuclein has been implicated to function in distinct steps of the SV cycle. We and others have demonstrated that α-synuclein regulates different stages of SV endocytosis (SVE) (14, 15, 16). In addition, there is evidence of α-synuclein interacting with the exocytic SNARE, synaptobrevin-2 (17, 18, 19), regulating fusion pore dynamics (20), and clustering of SVs, postfusion (21, 22, 23). The preponderance of functional studies supports synucleins acting postfusion, in SVE (14, 15, 16, 20, 24, 25).

Biochemical evidence in cell lines and neurons supports interactions between α-synuclein and the protein machinery of endocytosis (Hsc70, clathrin, AP2, endophilin) (24, 26). In previous studies, we showed, using pHluorin imaging and cholera toxin labeling, that αβγ-synuclein KO hippocampal neurons (27) exhibit slower SVE kinetics (14). Similar results have been obtained by monitoring transferrin uptake in α-synuclein KO neurons, which showed reduced endocytosis (24). In addition, we demonstrated that the interaction between α-synuclein and synaptobrevin-2 observed by other researchers (17, 23, 28) is part of a larger complex, of which AP180 is a major component (14). AP180 is a brain-specific adapter of synaptobrevin-2 (29) and along with AP2, acts to initiate clathrin-coated pit (CCP) formation by recruiting clathrin to synaptic membranes (19, 29, 30, 31, 32). With this in mind, we hypothesized that α-synuclein could be impacting SVE through a mechanism that involves lipids, AP180, and clathrin. Here, we aim to gain molecular insights regarding this mechanism by performing biochemical experiments with synthetic and physiological membranes as well as brain-derived clathrin-coated vesicles (CCVs).

Our experiments revealed that α-synuclein affects clathrin lattice size *in vitro*, and on physiological membranes, additionally, that AP180, α-synuclein, and clathrin colocalize and are arranged in a concentric pattern in larger clathrin puncta. Clathrin-α-synuclein colocalization is dependent on α-synuclein’s lipid-binding properties. We also show that the subsynaptic localization of α-synuclein is dynamic and can localize to the presynaptic plasma membrane, under conditions where bulk and SVE are promoted. Finally, we demonstrate that deletion of synucleins decreases brain-derived CCV perimeters. Collectively, these results indicate that α-synuclein may facilitate the growth of clathrin lattices on synaptic membranes.

## Results

### α-Synuclein affects the size of clathrin lattices on synthetic lipid monolayers

**Figure 1:**
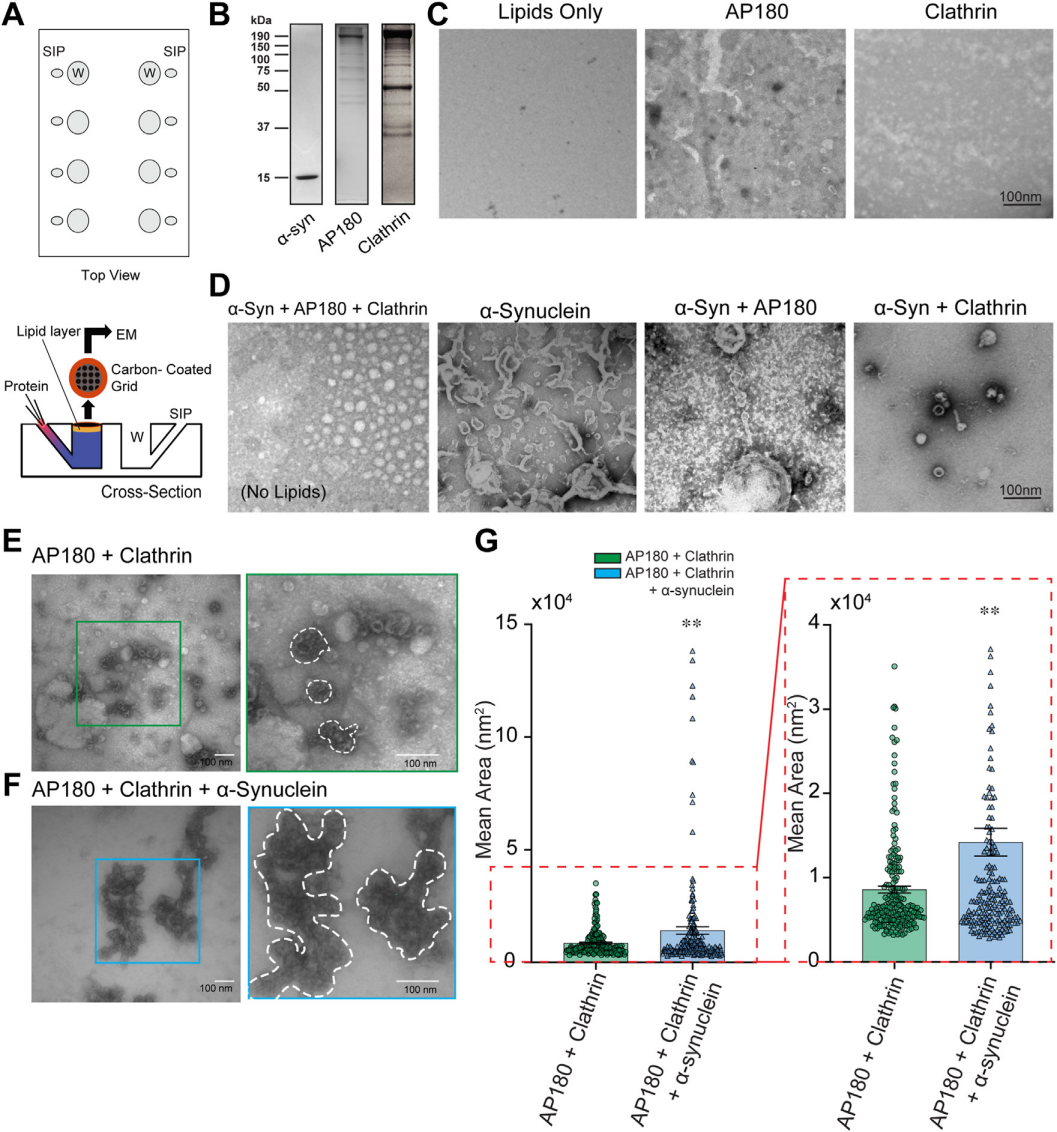
**Reconstitution of clathrin assembly using recombinant proteins.***A*, schematic of Teflon block and experimental setup used in monolayer reconstitutions. Wells (W) and side-injection ports (SIP) labeled. *B*, coomassie stain showing proteins used in reconstitution conditions. *C*–*E*, electron micrographs of constituent proteins added to lipid monolayers. *C*, electron micrographs of negative controls. From *left* to *right*: Lipids only, lipid monolayer with no added protein; AP180, recombinant AP180 added to lipid monolayer; Clathrin, brain-purified clathrin added to lipid monolayer. *D*, from *left* to *right*: all proteins without a lipid monolayer; α-Synuclein, recombinant WT mouse α-synuclein added to lipid monolayer; α-Syn+AP180, recombinant mouse AP180 and α-synuclein added to lipid monolayer; α-Syn+Clathrin, α-Synuclein and isolated clathrin added to lipid monolayer. *E*, electron micrographs of clathrin and AP180 added to lipid monolayer. Characteristic fullerene-shaped clathrin cages form. Inset: Zoomed image outlined in *green*. *F*, electron micrographs of α-synuclein, clathrin, and AP180 added to lipid monolayer. Inset: Zoomed image outlined in *blue*. Scale bar represents 100 nm. *G*, quantification of area of clathrin puncta obtained in conditions (*E*) and (*F*) includes expansion of data from 0 to 4 × 10^4^. Addition of α-synuclein increases the area of clathrin puncta. N = 3 independent experiments with at least ten micrographs per experiment. ∗*p* < 0.05; ∗∗*p* < 0.01. Scale bar represents 100 nm.

To explore the mechanisms by which α-synuclein regulates SVE, we investigated the effect of α-synuclein on AP180-dependent clathrin lattice formation on synthetic lipid membranes. We used a minimal component system for clathrin lattice assembly described by (32), in which a lipid monolayer containing PI(4,5)P2 is incubated, in a Teflon chamber, with different combinations of purified proteins in the presence of clathrin. In this lipid monolayer assay, AP180 alone is sufficient to recruit brain-purified clathrin (which contains small amounts of AP2) to the membrane and form clathrin lattices (32). We performed the lipid monolayer assay using the Teflon incubation chamber as shown in Figure 1*A*. We dropped the synthetic lipid mixture on top of the buffer in a well to form a monolayer, and after evaporation of the lipid diluent, chloroform, we placed a carbon-coated EM grid in the well face down onto the lipids (Fig. 1*A*). We purified recombinant mouse α-synuclein (14 KDa), rat AP180 (190 KDa) from *E. Coli*, and clathrin from pig brain (130 KDa heavy and 30–35 KDa light chains) (Fig. 1*B*). We injected these proteins in different combinations into the incubation chamber beneath the monolayer (Fig. 1*A*). After incubation, the grids were lifted from the chamber and processed for negative staining and EM. Representative micrographs show that the addition of clathrin-only has no effect on membranes due to its inability to directly bind them (Fig. 1*C*), while AP180 can bind the lipid monolayer and modestly deform it (Fig. 1*C*). Injection of recombinant mouse α-synuclein alone or in simple combination with AP180 or clathrin-only resulted in membrane ruffles and deformations (Fig. 1*D*) consistent with its ability to bend membranes (33, 34, 35, 36). Addition of both AP180 and clathrin led to the formation of discrete clathrin lattices (Fig. 1*E*), confirming previous literature showing AP180 is sufficient for clathrin assembly (32). Inclusion of mouse α-synuclein (2 μM; endogenous concentration) in this protein mixture resulted in significantly larger clathrin lattices on the lipid monolayer (Fig. 1, *E*–*G*) (AP180+clathrin = 8607 ± 413 nm^2^, AP180+clathrin+α-synuclein = 15,569 ± 2131 nm^2^; *p* < 0.01). This observation suggests that α-synuclein can impact the size of clathrin lattices under conditions where the formation of clathrin lattices is dependent on AP180 (Fig. 1*E*).

**Figure 2:**
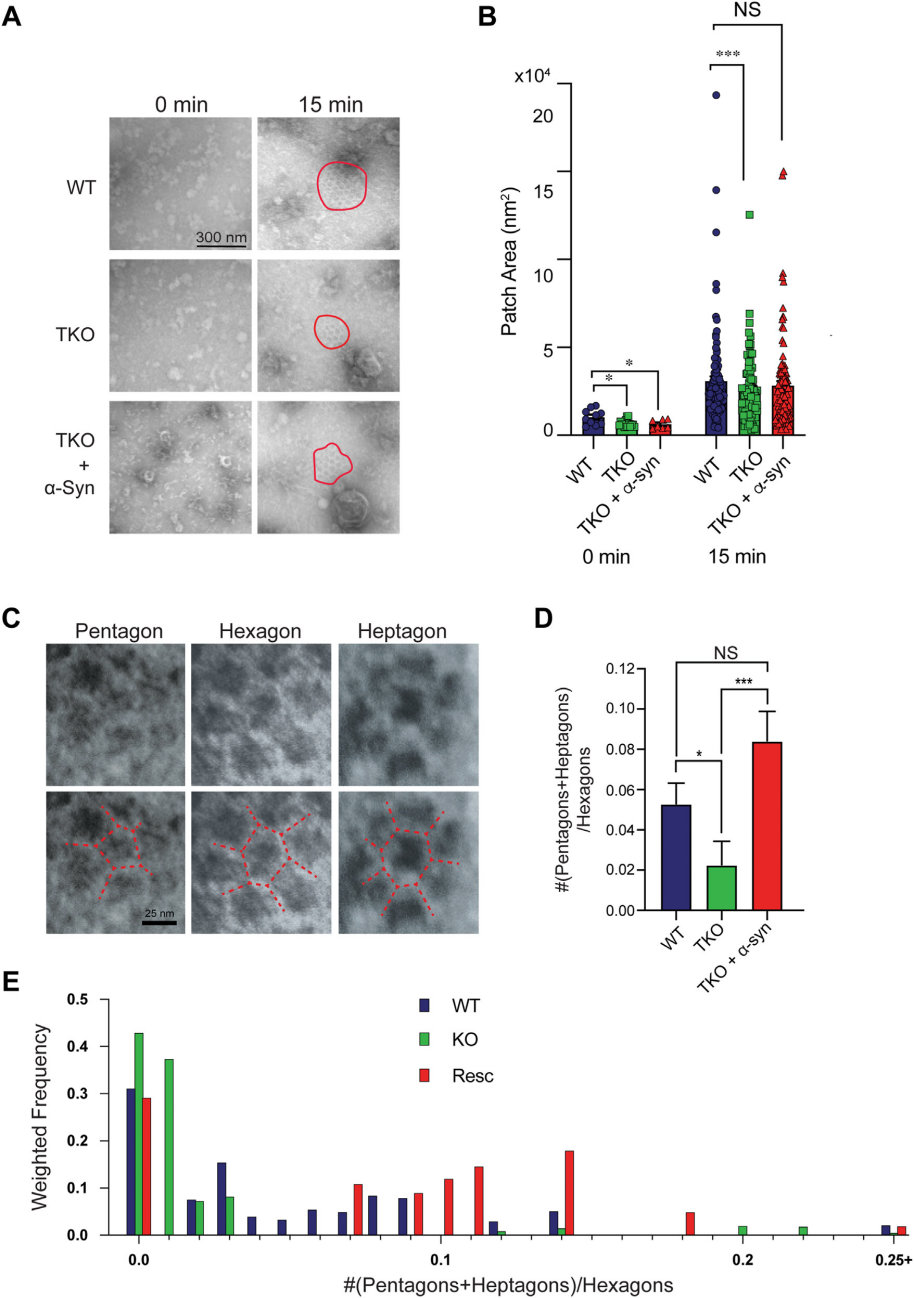
**Lipid monolayer assay to visualize clathrin recruitment from brain cytosol.***A*, electron micrographs of clathrin assembly into flat lattices and CCPs on membrane monolayers over time (0 and 15 min). Membrane monolayers were incubated with cytosol from WT, αβγ-synuclein KO (TKO), and mouse α-synuclein added to TKO cytosol (rescue). Clathrin puncta are highlighted with *red outlines*. N = 3 independent experiments with ten images per experiment. Scale bar represents 300 nm. *B*, comparison of clathrin patch areas in the three conditions mentioned above at 0, 15 min. *C*, examples of pentagon, hexagon, and heptagon clathrin lattices. Scale bar represents 25 nm. *D*, ratio of nonhexagons (pentagons and heptagons) to hexagon in clathrin lattices for each of the three conditions, t = 15 min. *E*, histogram of nonhexagon to hexagon ratio for the three conditions. The sum of all weights was used to normalize the data. For all graphs: ∗*p* < 0.05 and ∗∗∗*p* < 0.001. Welsh’s *t* test. TKO, triple knockout.

Next, we evaluated clathrin lattice assembly with mouse brain cytosol which contains the entire repertoire of soluble brain proteins and compared the effects of cytosol lacking α-synuclein. For this, we adapted the lipid monolayer assay for incubation with brain cytosol (Fig. 1*A*). (32). WT and an αβγ-synuclein triple knockout (TKO) brain cytosol were used to evaluate clathrin assembly into lattices. TKO cytosol was chosen as we previously showed that all synucleins are functionally redundant to regulate SVE (14). In these experiments, the lipid monolayer was incubated with brain cytosol (WT and TKO) in the absence (0 min) or presence of ATP (15 min) to monitor basal and active clathrin assembly, respectively. In the absence of ATP, we observed the formation of minute clathrin lattices, representing the baseline lattice size (Fig. 2, *A* and *B*, 0 min). The baseline assembly of clathrin structures, albeit very small, was significantly smaller when the membranes were incubated with TKO cytosol and was not changed by the addition of mouse α-synuclein (Fig. 2, *A* and *B*, t = 0, WT = 10,298 ± 1370 nm^2^ and TKO = 6644 ± 527 nm^2^, *p* < 0.05, TKO + α-syn = 6229 ± 942 nm^2^, *p* < 0.05).

Next, we tested clathrin assembly in the presence of an ATP reconstitution system for 15 min. We observed that incubation of the lipid monolayer with WT cytosol led to robust assembly of clathrin lattices (Fig. 2, *A* and *B*, WT, 15 min), in agreement with previous publications (32, 37, 38). The size of individual clathrin lattices were much larger and are quantified in Figure 2*B* (compare t = 0 and 15). The absence of α-synuclein resulted in significantly smaller clathrin lattices than WT (t = 15, WT = 37,204 ± 2648 nm^2^ and TKO = 25,624 ± 2049 nm^2^, *p* < 0.001). Supplementation of TKO cytosol with mouse α-synuclein (2 μM; at endogenous concentrations) partially rescued the size of the clathrin lattices formed on the monolayer after 15 min incubation with an ATP reconstitution system (Fig. 2*B*, t = 15, WT = 37,204 ± 2648 nm^2^ and t = 15, TKO+α-syn = 30,783 ± 2379 nm^2^, NS). This *in vitro* observation that addition of α-synuclein can rescue the reduced size of clathrin lattices formed on synthetic monolayers with TKO cytosol confirms the α-synuclein dependence of these effects.

Next, we examined the curvature of clathrin lattices formed by analyzing the fractions of pentagons and heptagons to hexagons in the clathrin formations (Fig. 2*C*). The presence of pentagons and/or heptagons indicates that a curved lattice is being generated (39, 40). We found that incubation with TKO cytosol leads to fewer nonhexagonal clathrin formations (Fig. 2, *D* and *E*) as compared to WT, indicating flatter clathrin lattices. The addition of recombinant mouse α-synuclein (2 μM) to TKO cytosol resulted in a greater number of lattices with pentagons and heptagons being present, shapes which are associated with curved lattices (Fig. 2, *D* and *E*).

Taken together, these two complementary *in vitro* experiments show an effect of α-synuclein on the size and curvature of clathrin/AP180 lattices formed on synthetic membranes suggesting that α-synuclein may play a direct role in the regulation of clathrin lattices.

### α-Synuclein localizes to large clathrin lattices on cell membranes

**Figure 3:**
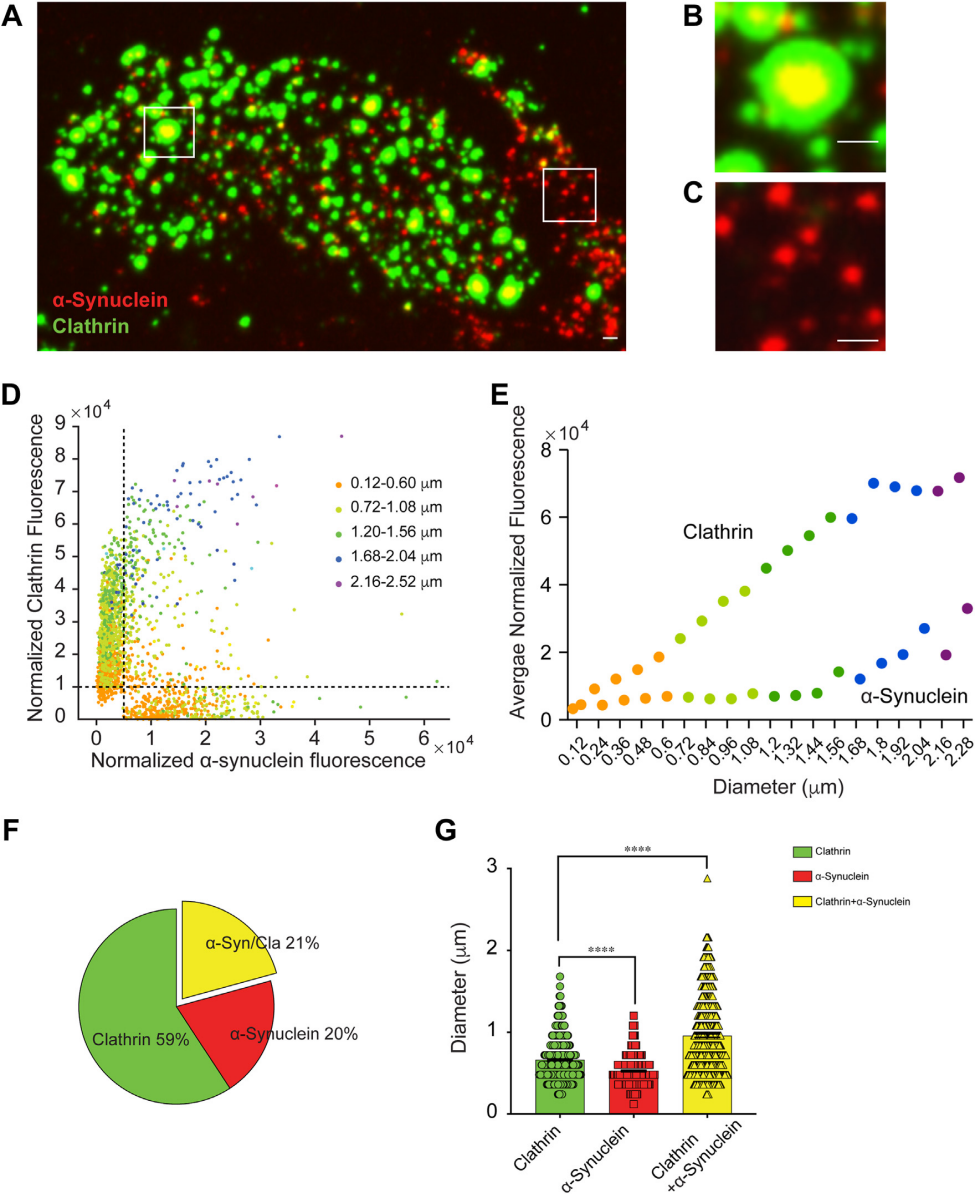
**α-Synuclein and clathrin colocalize in puncta on the plasma membrane.***A*, membrane sheets from PTK2 cells immunostained for α-synuclein (*red*) and clathrin heavy chain (*green*). α-Synuclein and clathrin are present in puncta on the membrane, with some puncta containing both proteins. Scale bar represents 1 μm. *B*, enlarged view of a clathrin+α-synuclein puncta. Note that α-synuclein localizes to the center of the clathrin patch. *C*, enlarged view of α-synuclein–only puncta. *D*, plot of fluorescence of clathrin *versus* α-synuclein, colored by size of the clathrin puncta. The *dashed lines* (x = 0.5 × 10^4^ and y = 1 × 10^4^) indicate the cut-offs used to demarcate puncta into clathrin-only (x < 0.5, y > 1), α-synuclein–only (x > 0.5, y < 1), and clathrin+α-synuclein (x > 0.5, y > 1) puncta. Note that clathrin and α-synuclein colocalize predominantly in larger size puncta. *E*, intensity of clathrin and α-synuclein in different puncta sizes. *F*, percent of the three populations of puncta observed on the membrane. Clathrin-only (*green*), α-synuclein–only (*red*), and clathrin+α-synuclein (*yellow*; abbreviated α-syn/Cla). *G*, average size of clathrin-only, α-synuclein–only, and clathrin+α-synuclein puncta. N = 3 independent experiments. ∗∗∗∗*p* < 0.0001. Scale bar represents 1 μm.

To test whether α-synuclein can affect clathrin lattices in a physiological context, we imaged α-synuclein localization relative to clathrin structures on cell membranes. We prepared adherent membranes by unroofing PTK2 (*Potorous tridactylus* epithelial kidney) cells grown on coverslips transfected with a construct expressing membrane-tethered GFP. Membranes were washed with cytosolic buffer, immunostained for clathrin heavy chain and α-synuclein, and imaged by confocal microscopy. Clathrin and α-synuclein were present in puncta on PTK2 membranes (Fig. 3, *A*–*C*), similar to observations in other cell types (24, 41, 42). A significant fraction of these puncta contained both proteins. All such puncta displayed a distinctive pattern of a clathrin puncta with α-synuclein colocalized at its inner core (Fig. 3*B*). Puncta containing only α-synuclein overall were smaller and more homogeneous in size (Fig. 3*C*).

To quantify the puncta, we measured fluorescence intensity and size of a linear region of interest (ROI) (cross-section) drawn across clathrin and α-synuclein puncta for all puncta on the membranes (Fig. 3*D*). Then, we plotted the relative clathrin fluorescence (Y-axis) *versus* the relative α-synuclein fluorescence (X-axis) for all puncta (n = 2071). We set cut-offs (x = 0.5 × 10^4^ and y = 1 x10^4^) by visually identifying two populations of puncta on the plot where the relative fluorescence of clathrin and α-synuclein were independent of each other (dashed lines, Fig. 3*D*). Using these cut-offs, we classified the total population of puncta into three categories: clathrin-only, α-synuclein-only, and clathrin+α-synuclein puncta. We classified 59% as clathrin-only, 20% of puncta as α-synuclein-only, and 21% as clathrin+α-synuclein under steady state conditions (Fig. 3*F*). The average diameters for clathrin-only, α-synuclein-only, and clathrin+α-synuclein puncta were 0.66 μm, 0.53 μm, and 0.96 μm, respectively (Fig. 3*G*; n = 1174, *p* = 0.001), indicating that puncta where clathrin+α-synuclein are localized together are consistently larger than clathrin-only and α-synuclein-only puncta.

To understand the relationship between α-synuclein and clathrin puncta size in steady state conditions, we binned the observed puncta into five size categories based on the diameter of clathrin puncta (orange: 0.12–0.60 μm, light green: 0.72–1.08 μm, dark green: 1.20–1.56 μm, blue: 1.68–2.04 μm, and purple: 2.16–2.52 μm) (Fig. 3*D*). While the measured diameters are an approximation—as the size measurement is distorted by the use of primary and dye-conjugated secondary antibodies, in addition to the resolution limitations of light microscopy—they are a useful measure to compare populations within the same experimental conditions. We plotted α-synuclein and clathrin intensity as a function of clathrin diameter, color coding the relative size categories (Fig. 3*E*). The average clathrin fluorescence exceeded that of α-synuclein, suggesting that α-synuclein is found within clathrin puncta when they colocalize. Average clathrin fluorescence increased linearly with puncta size, as expected. However, average α-synuclein fluorescence was largely constant and increased only in very large puncta (diameter > 1.6 μm) (Fig. 3*E*, blue and purple). This imaging data suggested that α-synuclein is colocalized with clathrin, preferentially in the larger clathrin puncta on physiological membranes, congruent with our *in vitro* data (Figs. 1*F* and 2B).

**Figure 4:**
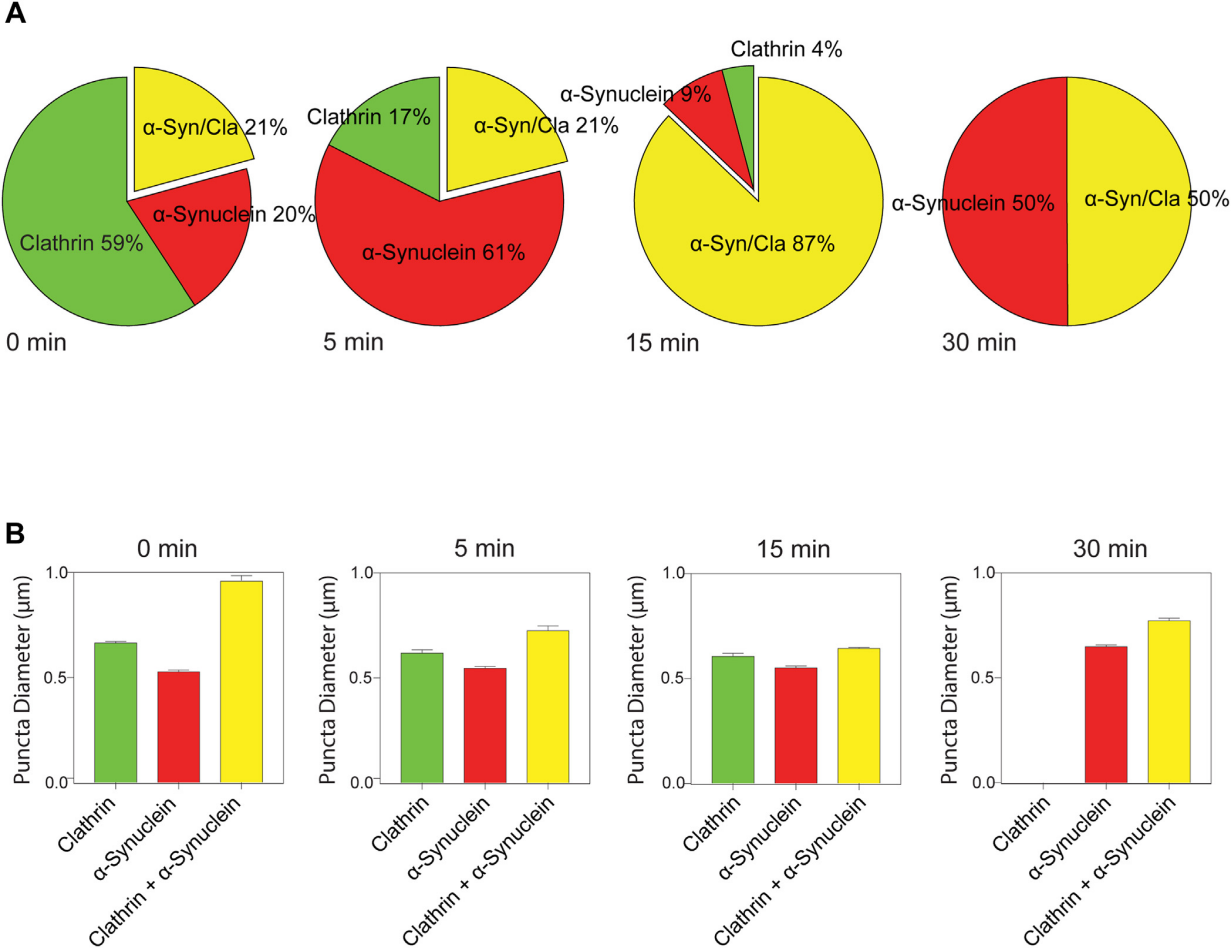
**Temporal changes of size and colocalization of α-synuclein and clathrin puncta.** PTK2 cells were unroofed, incubated with brain cytosol with an ATP regeneration system for the denoted time, and processed to detect α-synuclein and clathrin. *A*, fraction of the three types of puncta as a function of time. Clathrin-only (*green*), α-synuclein–only (*red*), and clathrin+α-synuclein (*yellow*; abbreviated α-syn/Cla). *B*, size comparisons of clathrin-only, α-synuclein–only, and clathrin+α-synuclein puncta at the denoted time points. N = 3 independent experiments with at least three cells analyzed per experiment. Total puncta analyzed per time: 0 min = 1982, 5 min = 636, 15 min = 4459, and 30 min = 1018.

Next, we incubated the PTK2 membranes with brain cytosol, an ATP regeneration system, and GTPγS to facilitate CCP formation and to test how the size and relative ratio of the three puncta classes changed over time (Fig. 4). Addition of GTPγS blocks dynamin function and leads to predominantly larger, flatter clathrin lattices. From the total population of puncta observed with incubation of brain cytosol, we observed that the fraction of clathrin-only puncta decreases over time (Fig. 4*A*: 59% at t = 0–0% at t = 30 min) with a concomitant increase in clathrin+α-synuclein puncta (Fig. 4*A*: 20% at t = 0–50% at t = 30 min), with the 5 min time point showing an intermediate level. This indicates that the localization of α-synuclein is dynamic and that it can be recruited to clathrin puncta. Comparing t = 15 and t = 30 time points suggests that α-synuclein can also come off clathrin puncta (Fig. 4*A*). The average size of α-synuclein-only and clathrin-only puncta remained constant, while the average clathrin+α-synuclein puncta diameter changed over time (Fig. 4*B*; 0.96 μm at t = 0 and 0.77 μm at t = 30). These data show that initially after unroofing cells, a fraction of α-synuclein is localized within the larger clathrin puncta on the membranes, which dynamically changes over time with the introduction of native brain cytosol proteins and ATP.

### Colocalization of α-synuclein and clathrin on the membrane depends on α-synuclein lipid/PI(4,5)P2 binding

**Figure 5:**
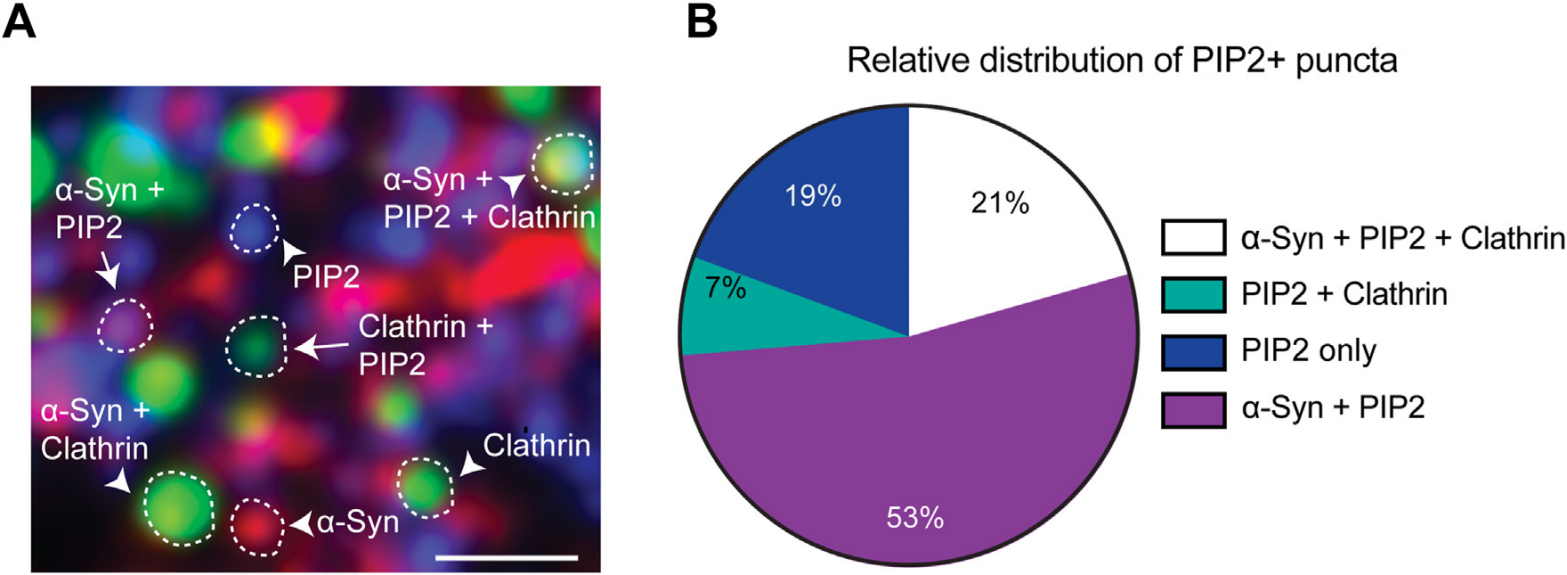
**α-Synuclein and PI(4,5)P2 colocalize on the plasma membrane.***A*, membrane sheets from PTK2 cells immunostained for α-synuclein (*red*), clathrin heavy chain (*green*), and PI(4,5)P2 (*blue*), with different classes of PI(4,5)P2 puncta highlighted (t = 0). *B*, relative distribution of α-synuclein and clathrin in the PI(4,5)P2 positive puncta. N = 2 independent experiments with four cells analyzed per experiment (3123 total puncta). Scale bar represents 1 μm.

Recently. it has been shown that α-synuclein colocalizes with PI(4,5)P2 in the membrane of many cell lines—A2780, HeLa, SH-SY5Y and SK-MEL-2 (43). To determine whether α-synuclein binds the membrane of PTK2 cells through PI(4,5)P2 when colocalized with clathrin, PTK2 membrane sheets (t = 0) were immunostained for clathrin heavy chain, α-synuclein, and PI(4,5)P2 and imaged by confocal microscopy (Fig 5*A*). As performed above (Figs. 3 and 4), we manually selected circular independent puncta. We set absolute thresholds for the three channels (threshold of pixel intensity: α-Syn = 5,000, PI(4,5)P2 = 5,000, clathrin = 6000) and classified the puncta into those containing individual constituents (clathrin, α-synuclein, or PI(4,5)P2 only) and two or more constituents. We quantified the relative distribution of clathrin and α-synuclein with respect to PI(4,5)P2 (n = 3123 puncta) (Fig. 5, *A* and *B*). We observed that the majority of the PI(4,5)P2 puncta quantified were positive for α-synuclein (53%), suggesting α-synuclein possibly stabilizes these puncta. Clathrin and α-synuclein also colocalized together with PI(4,5)P2 (21%). There was a small percentage of clathrin puncta that colocalized with PI(4,5)P2 (7%), which are probably bound *via* AP2. These results in conjunction with earlier results (24, 43) suggest that one mechanism through which α-synuclein affects clathrin lattice formation is through its interaction with PI(4,5)P2.

**Figure 6:**
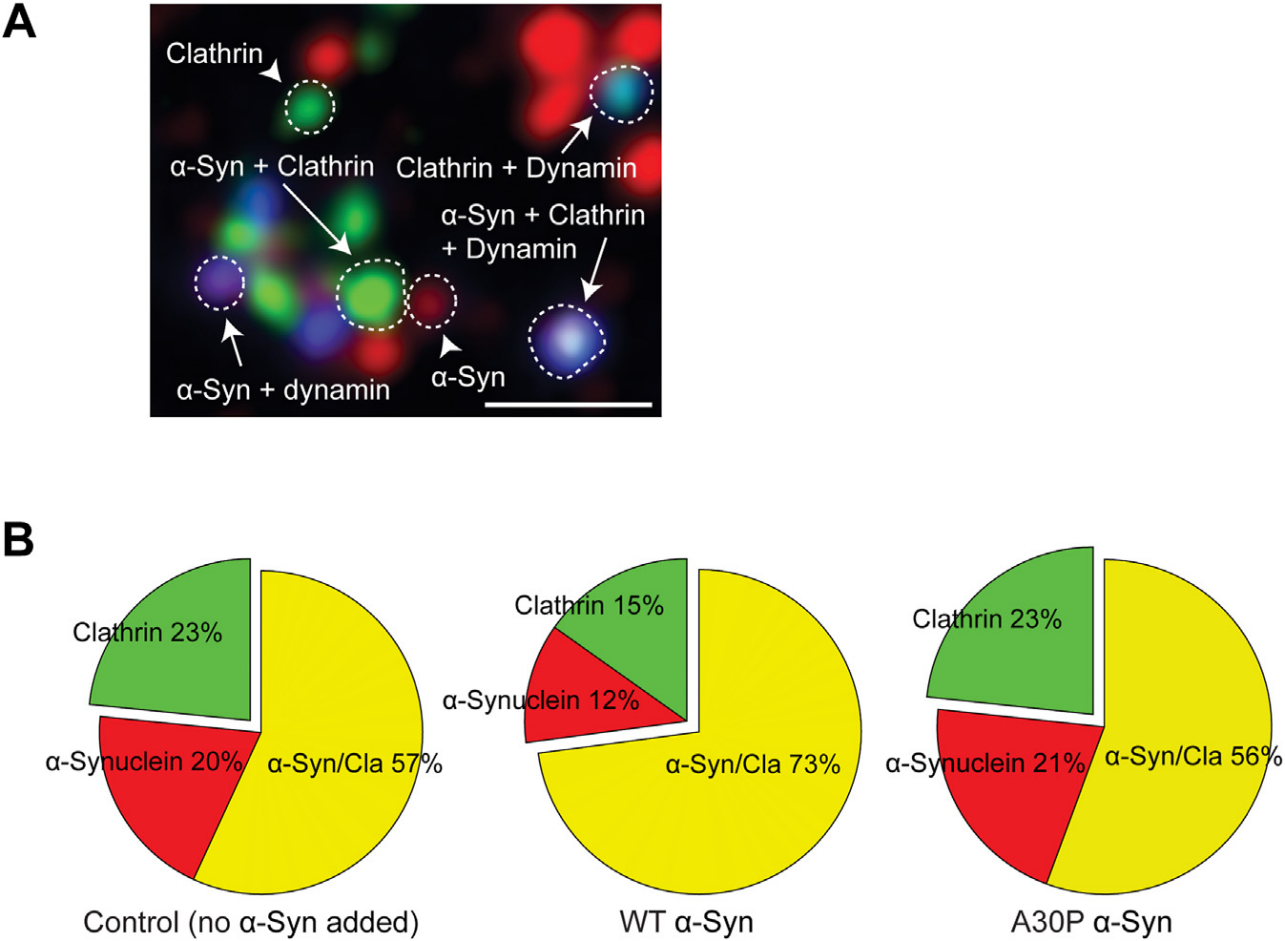
**α-Synuclein colocalization with clathrin on membranes depends on α-synuclein lipid binding.***A*, membrane sheets from PTK2 cells were immunostained for pan-dynamin (*blue*), clathrin heavy chain (*green*), and α-synuclein (*red*), with different classes of puncta highlighted, after incubation with no added α-synuclein (control), 4 μM WT human α-synuclein, and 4 μM human A30P α-synuclein for 15 min. *B*, relative distribution of α-synuclein and clathrin in dynamin-positive puncta in different conditions. N = 3 cells analyzed per experiment (ctrl = 3804 total puncta, WT = 4784 total puncta, A30P = 4271 total puncta). Scale bar represents 1 μm.

To test the effect of α-synuclein lipid binding on its colocalization with clathrin, we performed an experiment in which brain cytosol was supplemented with recombinant, monomeric human α-synuclein (4 μM) or membrane-binding deficient human A30P α-synuclein (4 μM). We incubated the membrane sheets with the supplemented cytosol for 15 min and stained for clathrin and α-synuclein (Fig. 6*A*). To ensure that successful invagination and vesicle formation occurred at 15 min, we performed immunostaining for dynamin in addition to clathrin and α-synuclein. We then quantified α-synuclein, clathrin, and α-synuclein/clathrin positive puncta that also colocalized with these dynamin-positive sites. We observed a larger fraction of puncta with both α-synuclein and clathrin when WT human α-synuclein was added for 15 min to brain cytosol compared to the control (WT α-syn 73% *versus* control 57%, Fig. 6*B*). However, when we added 4 μM human A30P α-synuclein to brain cytosol, we observed no change in the colocalization of α-synuclein and clathrin (A30P α-syn 56% *versus* control 57%, Fig. 6*B*). As A30P α-synuclein does not bind lipid membranes, this result suggests that α-synuclein binding to the membrane is necessary for its colocalization with clathrin.

### AP180 localizes at the center of the α-synuclein/clathrin large lattices on cell membranes

**Figure 7:**
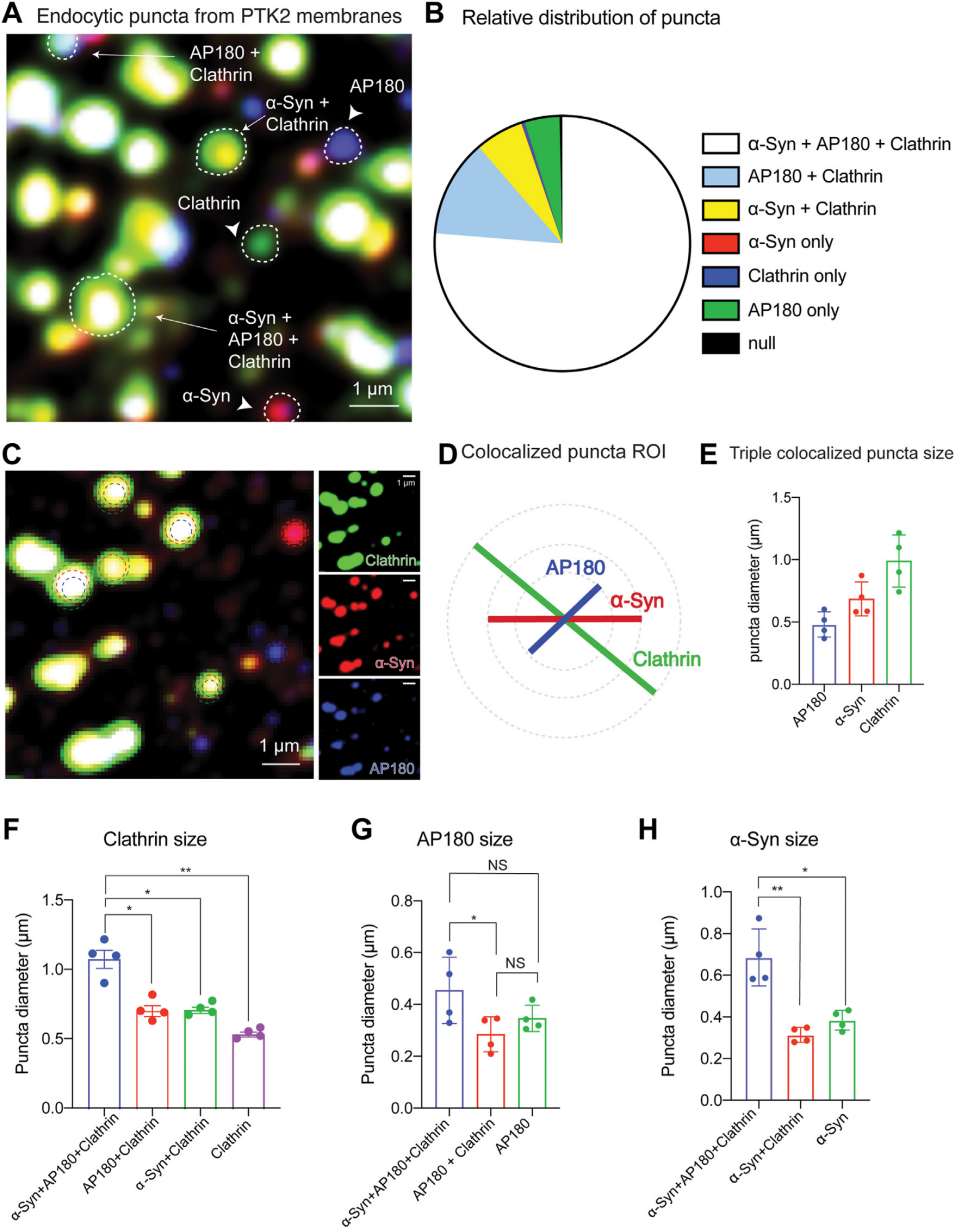
**AP180 colocalizes with α-synuclein and clathrin puncta on the plasma membrane.***A*, membrane sheets from PTK2 cells immunostained for α-synuclein (*red*), clathrin heavy chain (*green*), and AP180 (*blue*) different classes of puncta are highlighted. *B*, relative distribution of all observed puncta. α-synuclein+AP180+Clathrin puncta are the most prevalent. *C*, image showing α-synuclein+AP180+Clathrin puncta. AP180 localizes to the center of the clathrin puncta and is surrounded by α-synuclein, denoted by *dashed circles*. Insets show individual channels. *D*, scheme showing colocalization of clathrin, α-synuclein, and AP180. *E*, average diameter of AP180, α-synuclein, and clathrin in triple colocalized puncta. *F*, size of clathrin puncta in the denoted types of puncta containing clathrin. *G*, size of AP180 puncta in the three types of puncta containing AP180. *H*, size of α-synuclein puncta in the three types of puncta containing α-synuclein. N = 3 independent experiments, total number of puncta analyzed = 1681. ∗*p* < 0.05 and ∗∗*p* < 0.01; Scale bar represents 1 μm.

We extended the adherent membrane studies to include AP180, as it is a brain-specific clathrin adapter (44) and can transiently form a complex with α-synuclein (14). We incubated unroofed membranes with mouse brain cytosol, ATP reconstitution system, and GTPγS for 15 min, the time point we previously observed the maximum colocalization of clathrin+α-synuclein (Fig. 4*A*). We then immunostained for clathrin, α-synuclein, and AP180 and observed, as expected, heterogenous population of puncta (Fig. 7*A*) of varying sizes and protein content. We manually drew an ROI around each puncta and selected circular independent puncta. Then, we set absolute thresholds for the three channels (threshold of pixel intensity: α-Syn = 7000, AP180 = 6,000, clathrin = 6000) and classified the puncta into those containing individual proteins (clathrin, α-synuclein, or AP180-only) and two or more proteins (Fig. 7, *A* and *B*). After quantification (N = 4 experiments, 2637 puncta), we found six different types of puncta: clathrin+AP180+α-synuclein puncta (76.3%), clathrin+AP180 puncta (12.4%), clathrin+α-synuclein puncta (6.1%) clathrin-only puncta (4.4%), AP180-only puncta (0.4%), and α-synuclein-only puncta (0.1%) (Fig. 7*B*). We did not find AP180+α-synuclein puncta (0%) congruent with our previous biochemical data suggesting AP180 and α-synuclein do not directly interact (14).

We observed that the puncta containing all three proteins have a specific organization with AP180 in the center of the puncta, surrounded by a perimeter of α-synuclein within the clathrin pit (Fig. 7*C*). To quantify the concentric organization of these proteins, we measured the longest diameter of each circular puncta containing all three proteins (Fig. 7*D*). AP180 puncta which were located in the center had the smallest average diameter of 0.48 μm, with α-synuclein surrounding the AP180 puncta at an average diameter of 0.69 μm and with an outer clathrin shell diameter of 0.99 μm (Fig. 7*E*). Using the average diameters, we calculated the average area of each protein using the formula for the area of a circle (A = ***π***r^2^). We found that AP180 occupied on average 23% of the clathrin puncta, while α-synuclein occupied on average 49% of clathrin puncta. These data indicate that AP180 localizes to the center of the larger clathrin/α-synuclein puncta on physiological membranes and that there is a hierarchy of diameters with clathrin >α-synuclein >AP180.

To understand whether clathrin puncta size is different when AP180 or α-synuclein are present, we measured the clathrin puncta diameter depending on its colocalization with AP180 and α-synuclein. The diameter of the clathrin puncta in clathrin+α-synuclein+AP180 puncta were significantly larger (1.07 μm ± 0.1, *p* <0.05) than either clathrin+α-synuclein (0.71 μm ± 0.03) or clathrin+AP180 puncta (0.71 μm ± 0.55) and clathrin by itself (0.54 μm ± 0.02) (Fig. 7*F*). Next, we measured AP180 and α-synuclein puncta diameters within the various puncta classes. The diameter of AP180 puncta was larger in clathrin+α-synuclein+AP180 (0.45 μm ± 0.06) than clathrin+AP180 (0.28 μm ± 0.03) (Fig. 7*G*). The same pattern was observed for α-synuclein puncta diameter in the different classes (clathrin+α-synuclein+AP180: 0.69 μm ± 0.07 *versus* clathrin+α-synuclein: 0.31 ± 0.02, *p* < 0.05) (Fig. 7*H*). These puncta size comparisons suggest that each of the protein (AP180 and α-synuclein) puncta is larger when they are colocalized with one another and clathrin. These results strongly agree with our *in vitro* experiments suggesting α-synuclein and AP180 act synergistically to increase clathrin lattice size.

Our data thus far suggests that α-synuclein colocalizes with clathrin and influences the size of clathrin structures on both synthetic and cellular membranes through a molecular mechanism involving α-synuclein binding to membranes *via* PI(4,5)P2 and an indirect interaction with AP180. As α-synuclein is a presynaptic protein, it could affect the size of clathrin structures on synaptic membranes and so we investigated this possibility next.

### α-Synuclein is mobilized from SVs to synaptic membranes upon stimulation

**Figure 8:**
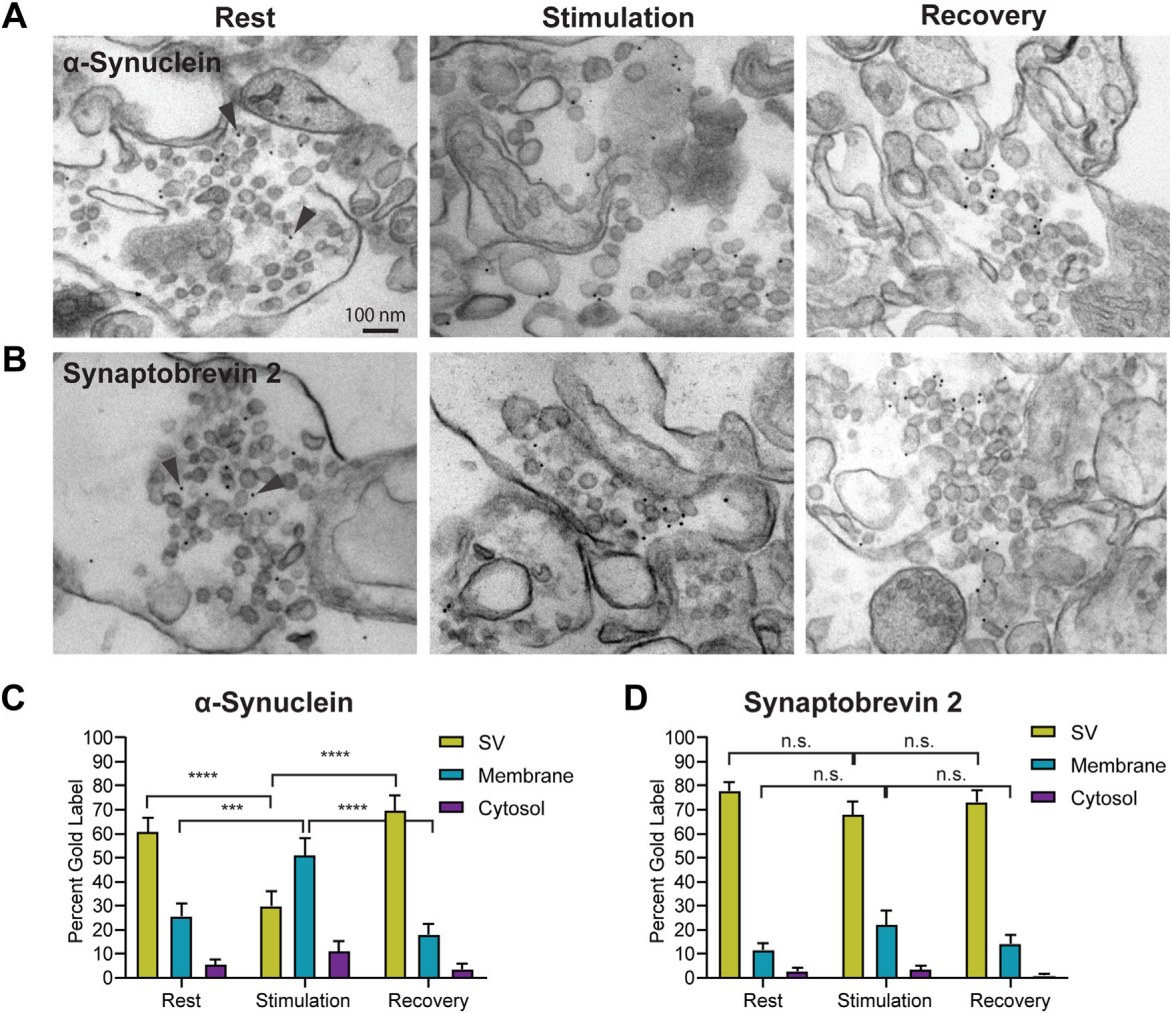
**α-Synuclein localization is dynamically regulated by neuronal activity.***A*, electron micrographs of permeabilized synaptosomes at rest, stimulated with 90 mM KCl, and upon recovery. Immunogold-labeled α-synuclein is localized to SVs at rest (*arrowhead*). During stimulation, α-synuclein disassociates from SVs and predominantly localizes to the synaptic membrane. During recovery, α-synuclein returns to the SV membrane. *B*, electron micrographs of permeabilized synaptosomes during rest, stimulation, and recovery conditions immunolabeled for synaptobrevin-2. Gold-labeled synaptobrevin-2 remains associated with the vesicle in all three conditions. *C* and *D*, quantification of gold label localization for α-synuclein and synaptobrevin-2. Bars represent percent gold label from n = 3 independent experiments for control conditions and n = 2 for remaining conditions. Twenty five to forty micrographs per condition were analyzed. ANOVA was used to determine significance. ∗∗∗*p* < 0.001 and ∗∗∗∗*p* < 0.0001. Scale bar represents 100 nm. SV, synaptic vesicle.

α-Synuclein is predominantly found on SVs at rest (22, 45, 46), consistent with its preferential binding to highly curved membranes (35, 36). Upon synaptic stimulation, fusion of SVs to the membrane increases the membrane surface area and decreases the number of SVs. To determine whether α-synuclein localization is altered upon stimulation and mobilized from SVs to synaptic membranes, we prepared synaptosomes from WT mouse brains and tested three conditions: rest, depolarization with high K+ stimulation, and repolarization. We then fixed the synaptosomes using a hypotonic fixative as described by previous studies (47). This technique fixes and breaks the synaptosomes simultaneously to increase the penetration of antibodies and capture the state of mobile proteins. The broken synaptosomes were immediately embedded in agarose to enable their manipulation for immunolabeling. We used monoclonal α-synuclein antibodies (clone42, BD Biosciences) that we previously showed by immunogold EM of α-synuclein KO synaptosomes to be highly specific (Fig. 8) (22). After incubation with gold-labeled secondary antibodies, the samples were embedded and processed for EM. Gold particles within a 9 nm radius of a vesicle, or membrane, were assigned to these subcellular categories, while the remaining particles were assigned to the cytosol. At rest, the majority of α-synuclein is present on SVs (61.1% on SVs, 25.9% on synaptic membranes, 5.9% in cytosol; Fig. 8, *A* and *B*). This is similar to the integral SV protein synaptobrevin-2 (78.2% on SV, 12.0% on membranes, 3.1% in cytosol; Fig. 8, *A* and *C*), in agreement with previous publications (22, 48). However, upon stimulating synaptosomes with high K^+^, α-synuclein relocates from SVs to the synaptic plasma membrane and cytosol (30.1% on SVs, 51.1% on membranes, 11.2% in the cytosol) This change was significant: α-synuclein on SVs at rest *versus* K^+^ stimulation, 61.1% *versus* 30.1%; *p* < 0.001; α-synuclein on membranes at rest *versus* K^+^ stimulation, 25.9% *versus* 51.1%; *p* < 0.001. This confirms previous confocal imaging which shows that α-synuclein becomes diffuse upon neuronal stimulation (48, 49, 50). In contrast, synaptobrevin-2 remains mainly on SVs when stimulated (rest *versus* K^+^ stimulation: 78.2% *versus* 68.4% on SVs; *p* = 0.095). Repolarization results in α-synuclein relocalization to the surface of SVs (70.1% on SVs, 18.2% on membranes, 3.8% in cytosol). The relocalization of α-synuclein was significant (K^+^ stimulation *versus* recovery: 30.1% *versus* 70.1% on SVs; *p* < 0.001). Thus, the subsynaptic location of α-synuclein is dynamic and is likely to be linked to the SV cycle. α-Synuclein localization on the synaptic plasma membrane upon neuronal stimulation supports the hypothesis that α-synuclein could localize with clathrin and AP180 in larger clathrin puncta at synaptic plasma membranes. Unfortunately, our attempts to double label clathrin or AP180 were unsuccessful, precluding us from showing that α-synuclein is recruited to CCPs. Overall, this experiment details the dynamic nature of α-synuclein’s localization and indicates that α-synuclein is likely to be on the plasma membrane, possibly through PI(4,5)P2 binding, allowing it to function in SVE.

### α-Synuclein affects the size of CCVs purified from brain

**Figure 9:**
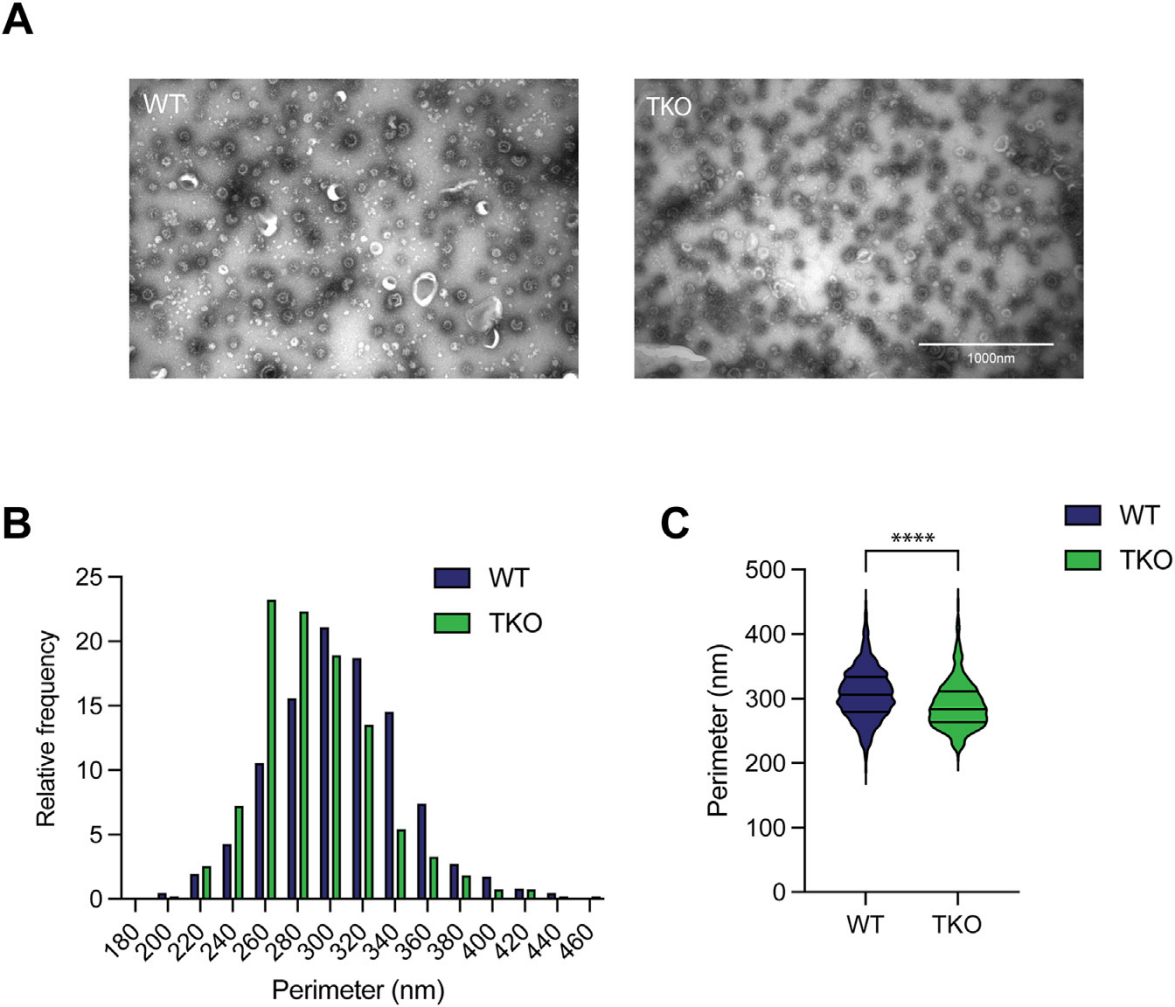
**Deletion of synucleins decreases clathrin-coated vesicle size.***A*, representative images of CCV purified from WT and αβγ-synuclein KO (TKO) mouse brain, negative stained with uranyl acetate for EM. Scale bar represents 1000 nm. *B* and *C*, frequency distribution and violin plots of clathrin-coated vesicle perimeters quantified from electron micrographs in panel A. (WT: Mean = 307.225 nm, 25% Percentile = 279.916, 75% Percentile = 333.48; TKO: Mean = 290.433 nm, 25% Percentile = 263.894, 75% Percentile = 311.599). Mann–Whitney U test was used to determine significance of difference in mean perimeters, U = 283,855, ∗∗∗∗ *p* < 0.0001. TKO, triple knockout.

To assess the role of α-synuclein in regulating the size of CCVs in the brain, we purified CCVs from both WT and αβγ-synuclein TKO mouse brains. Purified CCVs were dropped onto a grid and processed for EM (Fig 9*A*). CCVs were manually and independently measured by two blind raters. Their results were paired based on the distance between their centroids. In order to avoid quantifying arbitrary structures as true CCVs, ROIs without paired centroids within their radii were excluded from analysis. For each CCV, perimeter was averaged between the two raters and the results were plotted (Fig. 9*B*). A Mann–Whitney U test was performed to compare mean perimeter measurements between WT and TKO CCVs. The results indicated that the perimeter of WT CCVs was significantly greater than the perimeter of CCVs purified from TKO brain, U = 283,855, *p* <0.0001 (Fig. 9*C*). This suggests a regulatory role for synucleins in the formation of CCVs during their development on the membrane.

## Discussion

### α-Synuclein function and CCPs formation models

In this study, we show that α-synuclein increases the size of clathrin structures *in vitro* and colocalizes preferentially with larger clathrin structures on physiological membranes. Our endocytic reconstitution experiments (Figs. 1 and 2) demonstrate that recombinant α-synuclein can directly impact clathrin lattice formation in a minimal system, providing the first proof that α-synuclein could affect early steps of SVE. All previously published data inferred a role for α-synuclein in SVE through knockout and/or overexpression studies. Our cell membrane–based assays and CCV experiments indicate that α-synuclein impacts clathrin lattice size (Figure 3, Figure 4, Figure 7 and 9).

The clathrin structures we observe in our cell membrane assays are likely enlarged CCPs, whose size are increased because of membrane tension, unroofing, and the inclusion of GTP-γ-S to block dynamin function. However, the clathrin puncta also resemble flat clathrin lattices (also known as plaques), observed in a variety of cell lines, based on their larger area (>500 nm) (42, 51). Flat clathrin lattices are long-lived (42, 51, 52), though they turnover by budding CCPs at the edges (52) or bending the entire lattice (42). Thus, our findings suggest that α-synuclein could be involved in regulating the size of both CCP and flat clathrin lattices.

Presently, there are two main models to describe the growth of clathrin structures and the formation of CCPs: (1) the constant curvature model and (2) the constant area model. The constant curvature model proposes that curvature of CCPs develops, while endocytic protein adapters recruit and assemble clathrin triskelia to directly bend the plasma membrane, that is, curvature and clathrin assembly occur simultaneously. Consequently, a nucleation spot serves as a hub to develop a dome-like structure resembling a “rising sun”, which eventually is mechanically scissioned from the membrane by dynamin. In contrast, the constant area model suggests a flat clathrin lattice grows until it reaches sufficient size to bend and form a vesicle. Bending of the lattice would be achieved by late arrival of membrane-bending proteins. On membranes, a combination of the two models have been observed (40, 51, 53, 54, 55, 56). In this study, we show that inclusion of α-synuclein in the lipid monolayer assay increased both the size and curvature of the clathrin lattices formed *in vitro* (Fig. 2), in support of the constant curvature model. In fact, we find the number of nonhexagonal clathrin structures/area of lattice to be unaltered (Fig. 2*B* *versus* Fig. 2*D*), indicating they are changing in tandem. Furthermore, we find that α-synuclein and AP180 are present at the center of the clathrin puncta on membranes (Figs. 3 and 6). As α-synuclein can sense and generate membrane curvature (33, 35, 36), this supports the hypothesis that α-synuclein is regulating both the size and curvature of clathrin lattices simultaneously. Additional evidence comes from our observation that AP180, α-synuclein, and clathrin are organized in a concentric manner in clathrin puncta. This phenomenon has also been observed by Sochacki *et al.* (57), where endocytic proteins maintain characteristic nanodomains to ensure proper sorting and membrane bending. Additionally, we find that α-synuclein is recruited from brain cytosol to the center of clathrin puncta (Figure 3, Figure 4, Figure 5, Figure 6).

### Functions of α-synuclein with AP180, VAMP2, and PI(4,5)P2

Our data shows that α-synuclein affects clathrin lattice formation, and it colocalizes with AP180, clathrin, and PI(4,5)P2 on the membrane. This is consistent with the documented function of α-synuclein in SVE (14, 15, 16) where AP180 and clathrin are the main participants. Published studies have established that α-synuclein functionally interacts with clathrin machinery including clathrin heavy chain (CHC), AP2, AP180 (58, 59, 60). We have previously shown that α-synuclein is part of a protein complex with AP180 and VAMP2 (14). Our current data shows AP180 and α-synuclein colocalization in clathrin puncta, confirming the documented functional interactions. AP180 and VAMP2 have also been observed together in the center of clathrin lattices by Sochacki *et al.* (54). In those studies, AP180 and VAMP2 have a constrained distribution at the center of clathrin lattices and this is thought to underlie retrieval of the v-SNARE from the membrane. The AP180/VAMP2 distribution resembles our observations where α-synuclein, AP180, and clathrin are concentrically organized in large clathrin puncta. By extension, α-synuclein is also likely to participate in the retrieval of v-SNAREs from the membrane.

How do α-synuclein and AP180 affect clathrin puncta size? In Figure 7*F*, the size of clathrin structures is significantly larger only when colocalized with α-synuclein and AP180, suggesting that α-synuclein and AP180 cooperate to alter clathrin lattice size. This is further supported by the observations that α-synuclein and AP180 are never present individually in large clathrin structures. We hypothesize that when α-synuclein interacts with PI(4,5)P2 and AP180, the complex stabilizes clathrin triskelia on the membrane. This is supported by the data in Figure 1, where we showed that α-synuclein increases the size of clathrin lattices formed with AP180 and PI(4,5)P2 under minimal *in vitro* conditions.

While we see a significant fraction of clathrin puncta are colocalized with α-synuclein, a recent paper by Kaur *et al.* (41) showed that α-synuclein colocalizes with VAMP2 and syntaxin but not with CHC. This discrepancy is most likely due to the use of recombinant α-synuclein labeled with a dye and experimental conditions that did not include an ATP regeneration system. In our case, we investigated the localization of native α-synuclein on the membrane sheets which could explain the differences in our results. The labeled recombinant α-synuclein may not have been able to bind CHC in patches as they are already occupied by native α-synuclein. That aside, the observations by Kaur regarding VAMP2 colocalization with α-synuclein on membranes is consistent with reported literature, including our own observations on the αβγ-synuclein TKO model (22).

Recent publications have shown α-synuclein regulation of PI(4,5)P2 levels and clathrin-mediated endocytosis in cell lines and neurons (24). Increased α-synuclein levels correlate with increased PI(4,5)P2 levels and increased internalization of transferrin receptors and vice versa. In agreement, Jacob *et al.* (43) demonstrated α-synuclein is localized in subdomains of the membrane enriched in PI(4,5)P2. These findings align with our observations in Figure 5, wherein the majority of PI(4,5)P2-positive puncta colocalize with α-synuclein. Furthermore, addition of human WT α-synuclein increases colocalization of α-synuclein and clathrin, but the A30P mutant, which cannot bind lipids, fails to have this effect (Fig. 6). In combination, these data allow us to hypothesize a mechanism by which PI(4,5)P2 levels might help to recruit α-synuclein and AP180 to the center of clathrin patches. This would be expected to promote an increase in the size and curvature of clathrin lattices that we have observed. However, the precise mechanism for α-synuclein’s role in endocytosis through PI(4,5)P2/AP180/clathrin requires further exploration in intact neurons and synapses.

### α-Synuclein, clathrin-mediated endocytosis, and other endocytosis pathways in neurons

In previous work, we showed that α-synuclein regulates SVE (14). We tested different stimulation paradigms on αβγ-synuclein TKO that trigger different endocytic pathways. We observed endocytic deficits with electrical stimulation (100AP, 20 Hz) that triggers clathrin-mediated endocytosis and K^+^ stimulation that triggers bulk endocytosis. We went on to show by cholera toxin labeling and EM that synucleins regulate the kinetics of clathrin-mediated endocytosis. In this study, we discovered that α-synuclein dynamically moves from the SVs to the synaptic membranes in conditions that support bulk endocytosis. The large cisternae generated in bulk endocytosis can be part of the synaptic membranes we classified in Figure 8. Our results show evidence of regulation of large clathrin lattice formation *in vitro* and colocalization of α-synuclein, AP180, and clathrin on larger clathrin puncta aligning very well with the recent observation of large clathrin lattice formation on bulk endosomes (61, 62). Another interesting internalization pathway for SVs is ultra-fast endocytosis which has been recently described in the literature (63), yet there is no evidence of α-synuclein participation. We think investigating this avenue will be of great interest for the field.

Finally, we hypothesize that in pathological conditions, large clathrin lattices can be promoted by excess α-synuclein in the cell, comparable to our experiment in Figure 6, where an acute increase in α-synuclein levels increased the colocalization with clathrin. Stability and longevity of these large clathrin structures could lead to the deficit in internalization of SVs, an effect observed by Busch 2014 (15), where an acute overexpression of human α-synuclein blocked SVE. Together, these data open up new avenues for exploration, where α-synuclein, PI(4,5)P2, AP180, and clathrin function together in SVE and neurodegeneration.

## Experimental procedures

### Mice

WT and TKO mice were used in these studies. All experimental protocols involving animals were approved by the Institutional Animal Care & Use Committee at Yale University.

### Lipid monolayer assay with purified proteins

An 8-well Teflon block was arranged in a humid chamber and wells were filled with 40 to 60 μl of HKM buffer (25 mM Hepes pH 7.4, 125 mM potassium acetate, 5 mM magnesium acetate, 1 mM DTT). Lipid mixture (10% cholesterol, 40% PE, 40% PC, and 10% PI(4,5)P2 to final concentration of 0.1 mg/ml in a 19:1 mixture of chloroform to methanol) was carefully pipetted onto the buffer in each well. The blocks were incubated in a humid chamber at room temperature for 1 h to evaporate methanol/chloroform. Carbon-coated copper grids were placed carbon-side down onto each well. Proteins were introduced *via* side-injection ports beside each well. Final protein concentrations per well were as follows: 2 μM AP180 and α-synuclein, 500 nM purified bovine/porcine clathrin. Grids were incubated for 60 min in a humid chamber at room temperature and then removed from the block and immediately negative stained with 1% uranyl acetate. After drying, grids were imaged on a Phillip CM10 transmission electron microscope at 46k, 80 kV. Clathrin lattices were outlined manually, and the areas were quantified in Image J. Nonhexagonal clathrin lattices could not be quantified due to the high contrast needed to visualize these structures in these experiments.

### Purification of proteins

α-Synuclein and AP180 were recombinantly expressed and purified from BL21 (DE3) *E. Coli* as previously described (32, 33, 64). Clathrin was purified from porcine or bovine brains by purifying CCVs and disassembling cages as described (37). For the monolayer experiments, we used porcine and bovine clathrin interchangeably with no significant difference.

### Lipid monolayer assay with mouse brain cytosol

Five mg/ml WT and αβγ-synuclein KO mouse brain cytosol was added to wells of the Teflon block. Lipid mixture (see above) was pipetted on top of the cytosol. Blocks were incubated in a humid chamber for 1 h to evaporate methanol/chloroform. Carbon-coated copper grids were placed on top of the wells, and an ATP regenerating system (see above) and 150 μM GTPγS were introduced through a side-injection port. Grids were incubated in a humid chamber at 37 °C for 0 and 15 min and then removed from the block and immediately negative stained as described above. Grids were imaged on a FEI Tecnai Biotwin at scope 42k, 80 kV. Clathrin lattices were outlined manually; the area and number of pentagons, heptagons and hexagons were quantified in Image J.

### Reconstitution of endocytosis in PTK2 cells

PTK2 cells were transfected to express palmitoylated GFP and cultured for 48 h in a Mattek dish to confluence. Membrane sheets were prepared by sonication, using 20% output power, 1 brief pulse on the center of the well. The sheets were washed gently with cytosolic buffer and used within 20 min. The membranes were incubated for 0, 5, 15, 30 min with a mix of 2 mg/ml cytosol, 1.5 mM ATP, ATP reconstitution system (16.7 mM phosphocreatine, 16.7 U/ml creatine phosphokinase), and 150 μM GTPγS. The reaction was stopped by gently washing two times with cytosolic buffer and immediately fixed with 4% paraformaldehyde for 15 min in PBS. Membrane-bound proteins were detected using appropriate primary (clathrin: abcam ab2731, α-synuclein: Everest Biotech EB11713/Cell Signaling D37A6, and AP180: SCBT LP2D11) and secondary antibodies (ThermoFisher AlexaFluor 488,546,633). The membrane sheets were covered with cytosolic buffer. Z stack images were captured using spinning disc confocal microscopy, and analyses were performed in ImageJ using Fiji. Z slices were summed (Sum Slice) to create a single comprised of all channels. For each circular structure observed on membranes of interest, linear ROIs were drawn manually to each diameter of the puncta to analyze for size and fluorescence intensity.

All puncta were identified using analyze particle function Fiji with 1.0% as threshold in dark background. Puncta that overlapped with each other or with unusual shapes exceeding Fiji's threshold function of circularity 1.0 were excluded from puncta analysis (65). To determine puncta identity, we quantified the mean fluorescence for each protein for all ROIs. To identify whether a ROI was positive for a protein, we compared the mean fluorescence value of that protein against a cutoff value. The thresholds we choose for each channel were determined visually by the investigator and cross-checked with control puncta positive for individual proteins.

Some modifications were made for the membrane recruitment experiments with protein supplementation (α-synuclein overexpression) and the triple immunostaining for PI(4,5)P2 experiments. PTK2 cells were cultured for 24 h before sonication, as GFP transfection was not performed. For α-synuclein overexpression experiments, 4 μM recombinant human WT or A30P a-synuclein was added with the reconstitution mixture. For triple immunostaining for PI(4,5)P2, primary antibodies consisted of clathrin Abcam ab2731, α-synuclein Cell Signaling D37A6, PI(4,5)P2 Echelon Biosciences Z-P045; secondary antibodies consisted of ThermoFisher AlexaFluor 568, 633 and Abcam AlexaFluor 488. For α-synuclein overexpression experiments, primary antibodies consisted of clathrin Cell Signaling D3C6, α-synuclein Everest Biotech EB11713, and dynamin BD Transduction Clone 41. In the absence of GFP transfection, cells were incubated with Biotium CellBrite Steady 405 membrane dye to visualize membranes following immunostaining.

### Cytosol purification

Mouse brains were removed and washed in washing buffer (23 mM Tris–HCl, pH 7.4, 320 mM sucrose) for 30 s. Two brains were homogenized at 2500 rpm in a 5 ml potter homogenizer with 2 ml of homogenization buffer (25 mM Tris–HCl, pH 8.0, 500 mM KCl, 250 mM sucrose, 2 mM EGTA, and 1 mM DTT), using ten strokes at 5000 rpm. The homogenate was transferred to a 3.5 ml ultracentrifuge tube and centrifuged at 160,000*g* for 2 h at 4 °C. A PD-10 column was equilibrated with 25 ml cytosolic buffer, the supernatant was added to the PD-10 column, and then eluted with 3.5 ml of cytosolic buffer (25 mM Hepes–NaOH, pH 7.4, 120 mM potassium glutamate, 2.5 mM magnesium acetate, 20 mM KCl, and 5 mM EGTA–NaOH, pH 8.0, filtered and stored at 4 °C, with 1 mM DTT added immediately before use). Protein concentration was quantified by bicinchoninic acid assay. Mini cOmplete protease inhibitor cocktail was added, and 100 μl aliquots were flash frozen in liquid nitrogen and stored at −80 °C.

### Immuno-EM of mouse brain synaptosomes

#### Synaptosome isolation

Brains from WT mice (n = 3) were harvested and washed in homogenizing buffer (0.32 M sucrose, 10 mM Tris–HCl pH 7.4, mini cOmplete protease inhibitor tablet). Cerebella were removed, hemispheres separated, and white matter removed before adding the remaining brain to 10 ml of homogenizing buffer. Brains were homogenized in a 55 ml Potter homogenizer at 80% power for eight strokes. Homogenate was centrifuged at 2330*g* for 4 min at 4 °C in a JA-20 rotor. Pellet (P1) was discarded, and supernatant (S1) was transferred to a 50 ml centrifuge tube. S1 was recentrifuged at 18,850*g* for 12 min at 4 °C. Supernatant (S2) was discarded and pellet (P2) was resuspended in 6 ml of homogenizing buffer. Gradients of Percoll solutions (from bottom to top: 2.5 ml 23%, 3 ml 10%, and 2.5 ml 3%) were prepared in 15 ml glass tubes. Two microliters of resuspended P2 was added to the top of each gradient, and tubes were centrifuged at 18,850*g* for 12 min at 4 °C. Synaptosome fractions were collected by Pasteur pipette.

#### Stimulation

Washing buffer Krebs 1 (140 mM NaCl, 10 mM Tris–HCl pH 7.4, 5 mM KCl, 5 mM NaHCO_3_, 1.3 mM MgSO_4_, 1 mM Na-phosphate buffer) was added to the collected synaptosomes, and sample was centrifuged at 18,850*g* for 12 min. Pellet was resuspended in 1.5 ml Krebs 1 and divided equally into three 1.5 ml microcentrifuge tubes labeled for rest, stimulation, and recovery. The tubes were centrifuged at 16,000*g* for 3 min. Each pellet was resuspended in 500 μl of washing buffer Krebs 2 (10 mM glucose, 1.2 mM CaCl2 in Krebs 1) and incubated in a 37 °C water bath for 15 min. The rest condition was left in the bath, while the stimulation and recovery conditions were each removed, supplemented to 90 mM KCl final concentration, rotated by hand, and returned to the bath for 2 min. Stimulation and recovery tubes were then spun at 16,000*g* for 2 min. The stimulation pellet was resuspended in 200 μl Krebs 1 and left on ice. The recovery pellet was resuspended in 500 μl of washing buffer Krebs 3 (10 mM Glucose, 1 mM EGTA, 1 phosSTOP tablet in Krebs 1) and returned to the bath for 10 min. The recovery and rest tubes were removed from the bath and spun at 16,000*g* for 2 min. Pellets were resuspended in 200 μl of Krebs 1. All conditions were transferred into separate 50 ml centrifuge tubes filled with 30 ml of hypotonic fixative solution (3% paraformaldehyde, 0.25% glutaraldehyde in 5 mM Na-phosphate buffer) and incubated on ice for 30 min.

#### Agarose embedding

The samples were centrifuged in their 50 ml tubes at 18,850*g* at 4 °C for 12 min. Each pellet was resuspended in 400 μl of 120 mM Na-phosphate buffer. One hundred eighty microliter aliquots of each sample were pipetted into glass test tubes and held on ice until mixed with agarose. Pasteur pipettes and glass slide frames were warmed in a 60 °C oven. 3% agarose solution was prepared in 5 mM Na-phosphate buffer, warmed to 95 °C until agarose dissolved, and kept in a 54 °C water bath. One hundred eighty microliters of agarose solution was pipetted over each broken synaptosome sample, while the tube was held in the 54 °C water bath and mixed by gentle pipetting with a Pasteur pipette. The agarose-embedded samples were pipetted into the warmed glass slide frames and solidified on ice. Once solid, the frames were disassembled, leaving a solid agarose gel sheet on one glass slide. The gel was cut into 3 mm^3^ cubes with a razor blade and washed from the slide with 120 mM Na-phosphate buffer into petri dishes.

#### Immunolabeling and EM preparation

Each condition’s cubes were divided into five wells of glazed porcelain well plates. Cubes were incubated for 30 min with 5% BSA in solution A (0.5 M NaCl, 0.02 M Na-phosphate buffer) at room temperature. BSA solution was removed, and each well was incubated overnight with 200 μl of primary antibodies in solution A at 4 °C. Cubes were washed with 5 changes of solution A over the course of 1.5 h. Solution A was removed from the wells and cubes were incubated with secondary antibodies in solution A and 5% BSA for 6 h at room temperature. Cubes were washed with 5 changes of solution A and incubated at 4 °C overnight.

Agarose samples were prepared for resin embedding, sectioned, and stained with 2% uranyl acetate and 1% lead citrate before imaging on a FEI Tecnai Biotwin scope at 48k, 80 kV.

#### CCV purifications

We purified CCVs from adult WT and TKO brains following previously published protocols (14). The CCVs were spread on an EM grid and imaged as described in the above section.

#### Measurement and analysis of CCV dimensions

CCVs were independently and blindly quantified by two raters in ImageJ. Each CCV was approximated as a circle, and area and perimeter were measured. After ROIs were created, ROIs were paired between raters based on the distance between their centroids. In order to avoid quantifying arbitrary structures as true CCVs, ROIs without paired centroids within their radii were excluded from analysis. For each CCV, perimeter was averaged between the two raters and the results were plotted.

### Statistics

All experimental analysis was done blinded to condition. Statistical analysis was performed by GraphPad Prism and R studio. Data is presented as average ± SEM.

## Data availability

All data are presented in the manuscript.

## Conflict of interest

The authors declare that they have no conflicts of interest with the contents of this article.
